# Supplementary material for: Measuring mechanical anisotropy of the cornea with Brillouin microscopy
Source: Nat Commun. 2022 Mar 15;13:1354. doi: 10.1038/s41467-022-29038-5 (PMC8924229; doi:10.1038/s41467-022-29038-5)
Supplement: Supplementary file 1 — Supplementary Information [file 41467_2022_29038_MOESM1_ESM.pdf]

Supplementary Materials for *Measuring mechanical anisotropy of the cornea with Brillouin microscopy*

Amira M. Eltony[1], Peng Shao[1,+], and Seok-Hyun Yun[1,2,*]

[1]Harvard Medical School and Wellman Center for Photomedicine, Massachusetts General Hospital, Boston, MA, 02114, USA
[2]Harvard-MIT Health Sciences and Technology, Cambridge, MA, 02139, USA
[+]Present affiliation: Reveal Surgical Inc., Montréal, QC, H2N 1A4, Canada
[*]syun@hms.harvard.edu

Composite model of a corneal lamella

Let $\sigma_i^{(k)}$ and $s_j^{(k)}$ denote the i-th stress and j-th strain elements, respectively, in Voigt notation. Here, '1' is for 'xx', '2' for 'yy', '3' for 'zz', '4' for 'yz', '5' for 'xz', and '6' for 'xy' for Cartesian coordinates used to describe the cornea (Fig. 1C). On the other hand, '1' is for '11', '2' for '22', '3' for '33', '4' for '23', '5' for '13', and '6' for '12' for the 123-coordinate system defined with respect to a single collagen fibril (Fig. 1A). $C_{ij}^{(k)}$ denotes the ij-th elastic modulus for material 'k', such that we have stress-strain relations of the form (Voigt notation):

$$\begin{bmatrix} \sigma_1^{(k)} \\ \sigma_2^{(k)} \\ \sigma_3^{(k)} \\ \sigma_4^{(k)} \\ \sigma_5^{(k)} \\ \sigma_6^{(k)} \end{bmatrix} = \begin{bmatrix} C_{11}^{(k)} & C_{12}^{(k)} & C_{13}^{(k)} & 0 & 0 & 0 \\ C_{12}^{(k)} & C_{22}^{(k)} & C_{23}^{(k)} & 0 & 0 & 0 \\ C_{13}^{(k)} & C_{23}^{(k)} & C_{33}^{(k)} & 0 & 0 & 0 \\ 0 & 0 & 0 & C_{44}^{(k)} & 0 & 0 \\ 0 & 0 & 0 & 0 & C_{55}^{(k)} & 0 \\ 0 & 0 & 0 & 0 & 0 & C_{66}^{(k)} \end{bmatrix} \begin{bmatrix} s_1^{(k)} \\ s_2^{(k)} \\ s_3^{(k)} \\ s_4^{(k)} \\ s_5^{(k)} \\ s_6^{(k)} \end{bmatrix} \tag{S1}$$

We define a coordinate system for the lamella in which the 1-direction is parallel to the fibril axis, and the 2- and 3-directions are orthogonal to it (Fig. 1). For Brillouin measurements at a 180° angle, light scatters from longitudinal elastic waves which have displacement only along the direction of wave propagation. For example, an elastic wave propagating in the 1-direction has $s_2^{(k)} = s_3^{(k)} = 0$ and hence $\sigma_1^{(k)} = C_{11}^{(k)} s_1^{(k)}$.

First, consider the case of loading parallel to the fibril axis (1-direction). The stress in the composite is the sum of the stresses in all of the fibrils ($\sigma_1^{(f)}$) and in the extrafibrillar matrix ($\sigma_1^{(m)}$), weighted by their volume fractions (law of mixtures):

$$\sigma_1^{(\text{lamella})} = \sigma_1^{(f)} V^{(f)} + \sigma_1^{(m)} (1 - V^{(f)}) \tag{S2}$$

If we assume that there is no slippage between the fibrils and the matrix, the strain of the fibers and the matrix must be equal

(the ‘isostrain rule’):

$$s_1^{(\text{lamella})} = s_1^{(f)} = s_1^{(m)} \tag{S3}$$

Hence, the longitudinal modulus $C_{11}^{(\text{lamella})}$ is given by:

$$C_{11}^{(\text{lamella})} = C_{11}^{(f)} V^{(f)} + C_{11}^{(m)} (1 - V^{(f)}) \tag{S4}$$

Likewise, in the case of loading perpendicular to the fiber axis, the fibrils and the matrix experience different strains, but equal stress (‘isostress rule’):

$$\frac{1}{C_{22}^{(\text{lamella})}} = \frac{V^{(f)}}{C_{22}^{(f)}} + \frac{(1 - V^{(f)})}{C_{22}^{(m)}} \tag{S5a}$$
$$\frac{1}{C_{33}^{(\text{lamella})}} = \frac{V^{(f)}}{C_{33}^{(f)}} + \frac{(1 - V^{(f)})}{C_{33}^{(m)}} \tag{S5b}$$

If we assume that the extrafibrillar matrix is isotropic, $C_{11}^{(m)} = C_{33}^{(m)}$, and that the fibers are radially symmetric, $C_{22}^{(f)} = C_{33}^{(f)}$, we obtain:

$$C_{11}^{(\text{lamella})} = C_{11}^{(f)} V^{(f)} + C_{11}^{(m)} (1 - V^{(f)}) \tag{S6a}$$
$$C_{22}^{(\text{lamella})} = C_{33}^{(\text{lamella})} = \frac{C_{11}^{(m)} V^{(f)} + C_{33}^{(f)} (1 - V^{(f)})}{C_{11}^{(m)} C_{33}^{(f)}} \tag{S6b}$$

So we see that an individual lamella is transverse isotropic with plane of symmetry $2-3$ (i.e. orthogonal to the fibril axis).

Model of an orthogonal stack of lamellae

To compute the properties of the bulk stroma, we sum over the contributions of all the lamellae. The axes of the collagen fibrils in successive lamellae typically lie along orthogonal meridians in the medial-lateral and superior-inferior directions [Meek *et al*. Prog. Retin. Eye Res. **20**, 95-137 (2001)]. Therefore, we model the stroma as a summation of layers with half oriented in the medial-lateral direction and half in the superior-inferior direction. For the stroma, we define a coordinate system (x, y, z) with the z-direction orthogonal to the cornea, and the x- and y-directions tangential (medial-lateral and superior-inferior). In the tangential plane $x - y$, the ‘isostrain rule’ applies similarly to Eq. S4, so by symmetry:

$$C_{xx}^{(\text{stroma})} = C_{yy}^{(\text{stroma})} = \frac{1}{2} C_{11}^{(\text{lamella})} + \frac{1}{2} C_{33}^{(\text{lamella})} \tag{S7}$$

The z-direction is orthogonal to the fiber axes in all lamellae, so:

$$C_{zz}^{(\text{stroma})} = C_{22}^{(\text{lamella})} = C_{33}^{(\text{lamella})} \tag{S8}$$

In this model, the cornea is transverse isotropic with plane of symmetry $x-y$.

The stress-strain relation of a transverse-isotropic material in the xyz coordinate system can be written as:

$$\begin{bmatrix} \sigma_{xx} \\ \sigma_{yy} \\ \sigma_{zz} \\ \sigma_{yz} \\ \sigma_{yz} \\ \sigma_{xy} \end{bmatrix} = \begin{bmatrix} C_{xx} & C_{xy} & C_{xz} & 0 & 0 & 0 \\ C_{xy} & C_{xx} & C_{xz} & 0 & 0 & 0 \\ C_{xz} & C_{xz} & C_{zz} & 0 & 0 & 0 \\ 0 & 0 & 0 & G_{yz} & 0 & 0 \\ 0 & 0 & 0 & 0 & G_{yz} & 0 \\ 0 & 0 & 0 & 0 & 0 & G_{xy} \end{bmatrix} \begin{bmatrix} s_{xx} \\ s_{yy} \\ s_{zz} \\ s_{yz} \\ s_{xz} \\ s_{xy} \end{bmatrix} \tag{S9}$$

Here, G_{yz} $(= G_{xz})$ and G_{xy} correspond to shear moduli in the yz- (xz-) and xy-plane, respectively. By symmetry, $C_{xx} - C_{xy} = 2G_{xy}$, and there are five independent parameters in the stiffness matrix.

Direction-dependent longitudinal modulus

For transverse isotropic materials, there is an analytic expression for the effective longitudinal modulus at an angle θ to the z-axis (assuming material plane of symmetry $x-y$):

$$C(\theta) = \frac{1}{2}\left[C_{xx}\sin^2(\theta) + C_{zz}\cos^2(\theta) + G_{yz} + D(\theta)\right] \tag{S10}$$

with $D(\theta) = \sqrt{\left[(C_{xx} - G_{yz})\sin^2(\theta) - (C_{zz} - G_{yz})\cos^2(\theta)\right]^2 + (C_{xz} + G_{yz})^2\sin^2(2\theta)}$ and mass density ρ. When $\theta = 0$, $C(0) = C_{zz}$, and at $\theta = \pi/2$, $C(\pi/2) = C_{xx}$. This range of possible $C(\theta)$ values characterizes the scale of the anisotropy of the tissue. We introduce the parameters α_{xx}, α_{xz}, and α_{yz}, defined as:

$$\alpha_{xx} = \frac{C_{xx}}{C_{zz}} - 1 \tag{S11a}$$

$$\alpha_{xz} = \frac{C_{xz}}{C_{zz}} - 1 \tag{S11b}$$

$$\alpha_{yz} = \frac{G_{yz}}{C_{zz}} \tag{S11c}$$

This allows us to rewrite the expression for the longitudinal modulus as follows:

$$C(\theta) = \frac{C_{zz}}{2}\left[(1+\alpha_{xx})\sin^2(\theta)+\cos^2(\theta)+\alpha_{yz}\right] \\ + \frac{C_{zz}}{2}\sqrt{\left[(1+\alpha_{xx}-\alpha_{yz})\sin^2(\theta)-(1-\alpha_{yz})\cos^2(\theta)\right]^2+(1+\alpha_{xz}+\alpha_{yz})^2\sin^2(2\theta)} \quad \text{(S12)}$$

For soft tissues like the cornea, the shear modulus G_{yz} is typically much smaller than the longitudinal moduli C_{zz}, so $\alpha_{yz} \ll 1$. Assuming that the anisotropy of the cornea is also relatively small, $\alpha_{xx}, \alpha_{xz} \ll 1$. We can expand Eq. S12 to first order in the small parameters α_{xx}, α_{xz}, and α_{yz}, which yields:

$$C(\theta) \approx C(0)[\,1+(2\alpha_{xz}+4\alpha_{yz})\sin^2(\theta)\cos^2(\theta)+\alpha_{xx}\sin^4(\theta)] \quad \text{(S13)}$$

where $C(0) = C_{zz}$ as before.

The anisotropic parameter α_{xx}

The small parameter α_{xx} (defined in Eq. S11a) characterizes the degree of anisotropy of a transverse isotropic material. For a single lamella, this anisotropic parameter, $\alpha_{11}^{(\text{lamella})}$, is given by:

$$\alpha_{11}^{(\text{lamella})} = \frac{C_{11}^{(\text{lamella})}}{C_{33}^{(\text{lamella})}} - 1 \quad \text{(S14a)}$$

$$= \frac{\left(C_{11}^{(f)}V^{(f)}+C_{11}^{(m)}(1-V^{(f)})\right)\left(C_{11}^{(m)}V^{(f)}+C_{33}^{(f)}(1-V^{(f)})\right)}{C_{33}^{(f)}C_{11}^{(m)}} - 1 \quad \text{(S14b)}$$

If we set $C_{11}^{(m)} = \beta_1 C_{33}^{(f)}$ and $C_{11}^{(f)} = \beta_2 C_{33}^{(f)}$, we obtain:

$$\alpha_{11}^{(\text{lamella})} = \frac{\left(\beta_2 V^{(f)}+\beta_1(1-V^{(f)})\right)\left(\beta_1 V^{(f)}+1-V^{(f)}\right)}{\beta_1} - 1 \quad \text{(S15)}$$

Using Eqs. (S7) and (S8), the anisotropic parameter α_{xx} for an orthogonal stack of lamellae is related to the anisotropy of each lamella:

$$\alpha_{xx}^{(\text{stroma})} = \frac{C_{xx}^{(\text{stroma})}}{C_{zz}^{(\text{stroma})}} - 1 = \frac{C_{11}^{(\text{lamella})}+C_{33}^{(\text{lamella})}}{2C_{33}^{(\text{lamella})}} - 1 \quad \text{(S16a)}$$

$$= \frac{1}{2}\alpha_{11}^{(\text{lamella})} \quad \text{(S16b)}$$

Tables of fit parameters for all porcine samples

$\Omega(0)$	$\alpha_{xx}^{(\text{stroma})}$	δ	R^2	Measurement location
5.537±0.001	0.139±0.007	0*	0.92	center
5.511±0.001	0.106±0.010	0*	0.78	center
5.510±0.002	0.085±0.025	0.004±0.007	0.78	center
5.507±0.001	0.100±0.020	0.016±0.008	0.93	center
5.524±0.003	0.072±0.007	0.024*	0.74	2 mm from center
5.515±0.001	0.108±0.018	0.018±0.007	0.96	2 mm from center
5.520±0.002	0.065±0.031	0.011±0.012	0.74	3 mm from center
5.501±0.002	0.119±0.024	0.015±0.008	0.93	3 mm from center
5.496±0.001	0.172±0.010	0*	0.90	3 mm from center
5.537±0.002	0.099±0.026	0.017±0.010	0.90	3 mm from center
5.527±0.002	0.138±0.026	0.005±0.007	0.89	4 mm from center

Table S1. Angle-dependence of porcine corneas *ex vivo*. Please refer to Fig. 4. Fitted value ± confidence interval for each parameter, coefficient of determination (R^2), and measurement location for the 11 *ex vivo* porcine corneas measured. The δ parameter was constrained to $0 < \delta < 0.25\,\alpha_{xx}^{(\text{stroma})}$. * indicates δ at bound.

Corneal ROC	$\Omega(0)$	$\alpha_{xx}^{(\text{stroma})}$	δ	R^2	Sample
8.72	5.567±0.001	0.120±0.010	0.040*	0.78	1 (∼normal)
8.64	5.563±0.002	0.082±0.005	0.027*	0.84	1 (tilted)
8.79	5.560±0.003	0.122±0.036	0.019±0.014	0.81	2 (tilted)
7.61	5.525±0.002	0.089±0.007	0.030*	0.79	3 (tilted)
7.89	5.532±0.002	0.075±0.006	0.025*	0.79	4 (tilted)
8.34	5.525±0.003	0.115±0.032	0.015±0.012	0.78	5 (tilted)

Table S2. Anisotropy in Brillouin maps of the porcine cornea *ex vivo*. Please refer to Fig. 6. Corneal radius of curvature (ROC) calculated using corneal surface coordinates, fitted value ± confidence interval for each parameter, coefficient of determination (R^2), and sample orientation for the 5 *ex vivo* porcine corneas measured. The δ parameter was constrained to $0 < \delta < 0.25\,\alpha_{xx}^{(\text{stroma})}$. * indicates δ at bound.

Table of fit parameters for the human subjects

$\Omega(0)$	$\alpha_{xx}^{(\text{stroma})}$	δ	R^2	Fixation angle
5.717±0.003	0.027±0.021	0.009*	0.09	0°
5.697±0.005	0.064±0.065	0.015±0.029	0.46	$\sim$ 20°
5.696±0.003	0.070±0.012	0.023*	0.60	$\sim$ 20°
5.700±0.004	0.052±0.010	0.017*	0.54	$\sim$ 20°

Table S3. Anisotropy of the human corneas *in vivo*. Please refer to Fig. 5. Fitted value ± confidence interval for each parameter, coefficient of determination (R^2), and eye fixation angle (relative to laser) for the 4 human subjects measured. A corneal radius of curvature (ROC) of 7.8 mm was used in calculating the beam incidence angles. The δ parameter was constrained to $0 < \delta < 0.25\,\alpha_{xx}^{(\text{stroma})}$. * indicates δ at bound.
