# Supplementary material for: Measuring mechanical anisotropy of the cornea with Brillouin microscopy
Source: Nat Commun. 2022 Mar 15;13:1354. doi: 10.1038/s41467-022-29038-5 (PMC8924229; doi:10.1038/s41467-022-29038-5)
Supplement: Supplementary file 3 — Reporting Summary [file 41467_2022_29038_MOESM3_ESM.pdf]

nature portfolio

Corresponding author(s): Seok-Hyun Yun

Last updated by author(s): Jan 21, 2022

Reporting Summary

Nature Portfolio wishes to improve the reproducibility of the work that we publish. This form provides structure for consistency and transparency in reporting. For further information on Nature Portfolio policies, see our Editorial Policies and the Editorial Policy Checklist.

Statistics

For all statistical analyses, confirm that the following items are present in the figure legend, table legend, main text, or Methods section.

n/a	Confirmed	
☐	☒	The exact sample size (n) for each experimental group/condition, given as a discrete number and unit of measurement
☐	☒	A statement on whether measurements were taken from distinct samples or whether the same sample was measured repeatedly
☒	☐	The statistical test(s) used AND whether they are one- or two-sided<br>*Only common tests should be described solely by name; describe more complex techniques in the Methods section.*
☐	☒	A description of all covariates tested
☐	☒	A description of any assumptions or corrections, such as tests of normality and adjustment for multiple comparisons
☐	☒	A full description of the statistical parameters including central tendency (e.g. means) or other basic estimates (e.g. regression coefficient) AND variation (e.g. standard deviation) or associated estimates of uncertainty (e.g. confidence intervals)
☒	☐	For null hypothesis testing, the test statistic (e.g. F, t, r) with confidence intervals, effect sizes, degrees of freedom and P value noted<br>*Give P values as exact values whenever suitable.*
☒	☐	For Bayesian analysis, information on the choice of priors and Markov chain Monte Carlo settings
☒	☐	For hierarchical and complex designs, identification of the appropriate level for tests and full reporting of outcomes
☒	☐	Estimates of effect sizes (e.g. Cohen's d, Pearson's r), indicating how they were calculated

Our web collection on statistics for biologists contains articles on many of the points above.

Software and code

Policy information about availability of computer code

Data collection	Data acquisition was performed using custom software written in Python 3.8.5 with the PyQtGraph 0.12.3 library.
Data analysis	Data analysis was performed using custom software written in Python 3.8.8 using the Spyder 5.2.1 environment. The SciPy 1.6.2 and Lmfit 1.0.2 libraries were used for non-linear least-squares minimization and curve-fitting.

For manuscripts utilizing custom algorithms or software that are central to the research but not yet described in published literature, software must be made available to editors and reviewers. We strongly encourage code deposition in a community repository (e.g. GitHub). See the Nature Portfolio guidelines for submitting code & software for further information.

Data

Policy information about availability of data

All manuscripts must include a data availability statement. This statement should provide the following information, where applicable:

- Accession codes, unique identifiers, or web links for publicly available datasets
- A description of any restrictions on data availability
- For clinical datasets or third party data, please ensure that the statement adheres to our policy

The processed data that support the findings of this study are included in this published article and its Supplementary Materials. Raw data are available from the corresponding author upon reasonable request.

Field-specific reporting

Please select the one below that is the best fit for your research. If you are not sure, read the appropriate sections before making your selection.

☒ Life sciences ☐ Behavioural & social sciences ☐ Ecological, evolutionary & environmental sciences

For a reference copy of the document with all sections, see nature.com/documents/nr-reporting-summary-flat.pdf

Life sciences study design

All studies must disclose on these points even when the disclosure is negative.

Sample size	Sample size was dependent on availability of tissue, and was considered sufficient when the sample-to-sample variability could be well-estimated.
Data exclusions	Angle-dependence data (as shown in Fig. 4) were acquired for 12 different porcine corneas, but 1 sample was excluded because the repeated scans were not consistent, likely indicating dynamic hydration changes which would confound the measurement. No other data exclusions were made.
Replication	All data acquired in this study, with the exception of the data exclusion noted above, is presented directly in the manuscript.
Randomization	This study only measured normal corneas and did not seek to find correlations between groups or with any particular characteristic, so randomization was not applicable.
Blinding	This study only measured normal corneas and did not seek to find correlations between groups or with any particular characteristic, so blinding was not applicable.

Reporting for specific materials, systems and methods

We require information from authors about some types of materials, experimental systems and methods used in many studies. Here, indicate whether each material, system or method listed is relevant to your study. If you are not sure if a list item applies to your research, read the appropriate section before selecting a response.

Materials & experimental systems

n/a	Involved in the study	
☒	☐	Antibodies
☒	☐	Eukaryotic cell lines
☒	☐	Palaeontology and archaeology
☒	☐	Animals and other organisms
☐	☒	Human research participants
☒	☐	Clinical data
☒	☐	Dual use research of concern

Methods

n/a	Involved in the study	
☒	☐	ChIP-seq
☒	☐	Flow cytometry
☒	☐	MRI-based neuroimaging

Human research participants

Policy information about studies involving human research participants

Population characteristics	There were 4 participants, 3:1 (M:F), 31.5 ± 2.4 Y/O. All had normal corneas. In this study, a `normal cornea' was classified as: less than 3 diopters refractive error, corneal thickness between 495 and 600 μm, normal corneal topography, no corneal pathology, and no history of eye disease.
Recruitment	Participants were recruited by their primary ophthalmologist who informed them of the possibility to participate in this research study. This study only measured normal corneas and did not seek to find correlations between groups or with any particular characteristic, so the influence of self-selection or other biases is likely minimal.
Ethics oversight	The Institutional Review Board (IRB) of Partners HealthCare,<br>The Partners Human Research Committee (PHRC),<br>The Institutional Review Board of IROC, Zürich,<br>The Woolfson Eye Institute Ethics Committee

Note that full information on the approval of the study protocol must also be provided in the manuscript.
